# Supplementary material for: Salvaging high-quality genomes of microbial species from a meromictic lake using a hybrid sequencing approach
Source: Commun Biol. 2021 Aug 23;4:996. doi: 10.1038/s42003-021-02510-6 (PMC8382752; doi:10.1038/s42003-021-02510-6)
Supplement: Supplementary file 2 — Supplementary Information [file 42003_2021_2510_MOESM2_ESM.pdf]

Supplementary Information

Salvaging high-quality genomes of microbial species from a meromictic lake using a hybrid sequencing approach

Yu-Hsiang Chen[1,2,3], Pei-Wen Chiang[3], Denis Yu Rogozin[4,5], Andrey G. Degermendzhy[4], Hsiu-Hui Chiu[3], Sen-Lin Tang[2,3*]

[1]Bioinformatics Program, Taiwan International Graduate Program, National Taiwan University, Taipei, Taiwan

[2]Bioinformatics Program, Institute of Information Science, Taiwan International Graduate Program, Academia Sinica, Taipei, Taiwan

[3]Biodiversity Research Center, Academia Sinica, Taipei, Taiwan

[4]Institute of Biophysics, Siberian Division of Russian Academy of Sciences, Krasnoyarsk, Russia

[5]Siberian Federal University, Krasnoyarsk, Russia

* Corresponding author

Biodiversity Research Center, Academia Sinica

E-mail: sltang@gate.sinica.edu.tw

Tel: +886-2-27893863

Fax: +886-2-27890844

Supplementary Figures

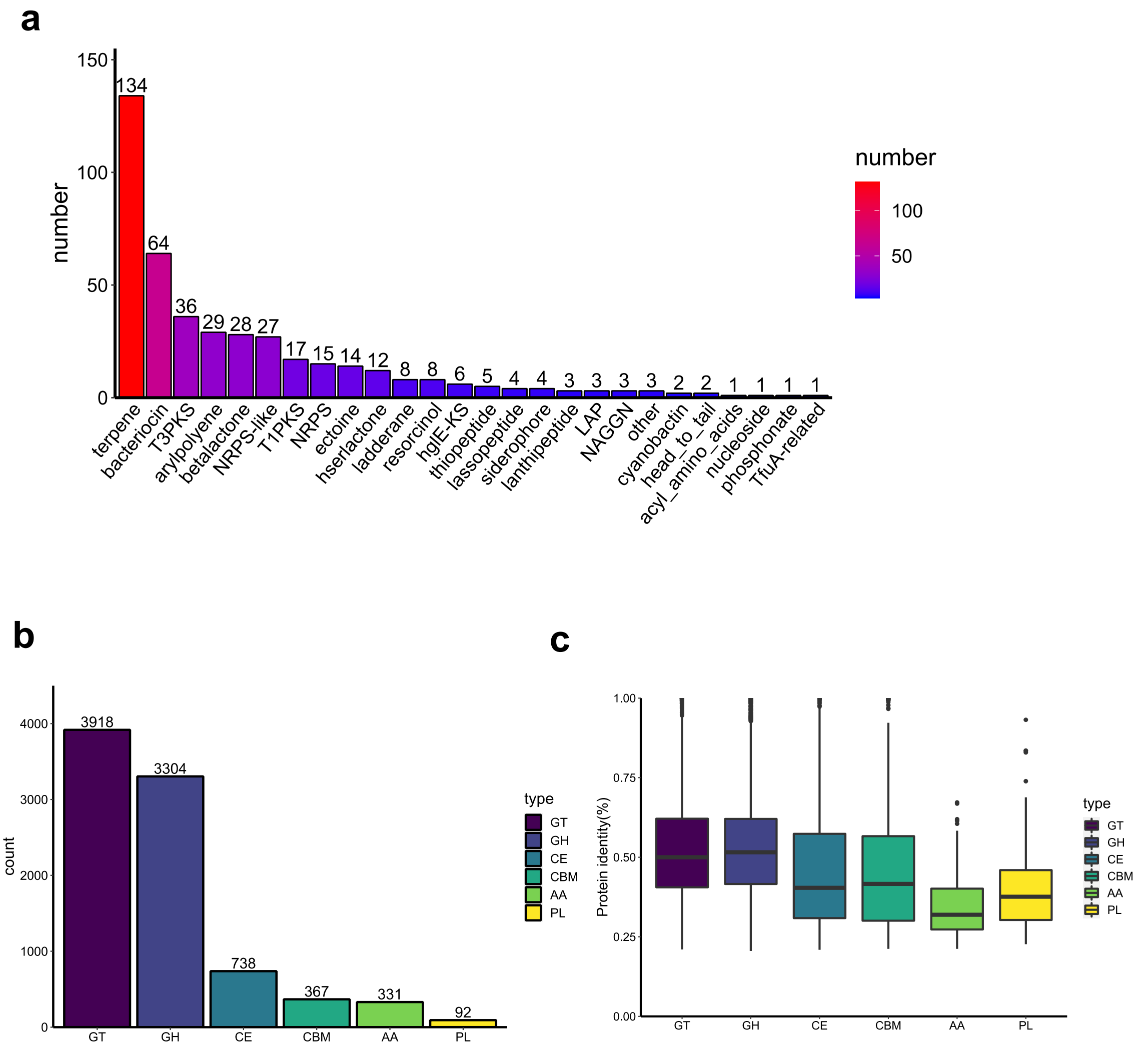

Supplementary Figure 1. Predicted secondary metabolite biosynthetic clusters and carbohydrate active-enzymes from recovered MAGs. a. The numbers of different types of BGCs identified from recovered MAGs by antiSMASH 5.0. The description of different types of secondary metabolite clusters can be found in the glossary from antiSMASH (https://docs.antismash.secondarymetabolites.org/glossary/). **b.** The numbers of different types of CAZymes identified from MAGs using dbCAN 5.0. **c.** Distribution of protein identities between newly identified CAZymes and their best hits in the NCBI nr database. The center line represents median, box limits represent upper and lower quartiles, whisker represents 1.5X interquartile range, and black dots represent outliers. Abbreviations in figure S1a: T3PKS, Type III Polyketide synthase

(PKS); NPRS, Non-ribosomal peptide synthetase cluster; hglE-KS, heterocyst glycolipid synthase-like PKS; LAP, Linear azol(in)e-containing peptides; NAGGN, N-acetylglutaminylglutamine amide; head_to_tail, Head-to-tail cyclised cluster; acyl_amino_acids, N-acyl amino acid cluster; TfuA-related; TfuA-related ribosomally synthesized and post-translationally modified peptides. Abbreviation in figure S2b: c:GT, GlycosylTransferase; GH, Glycoside Hydrolase; CE, Carbohydrate esterase; CBM, Carbohydrate-Binding Module; AA, Auxiliary Activity; PL, Polysaccharide Lyase.

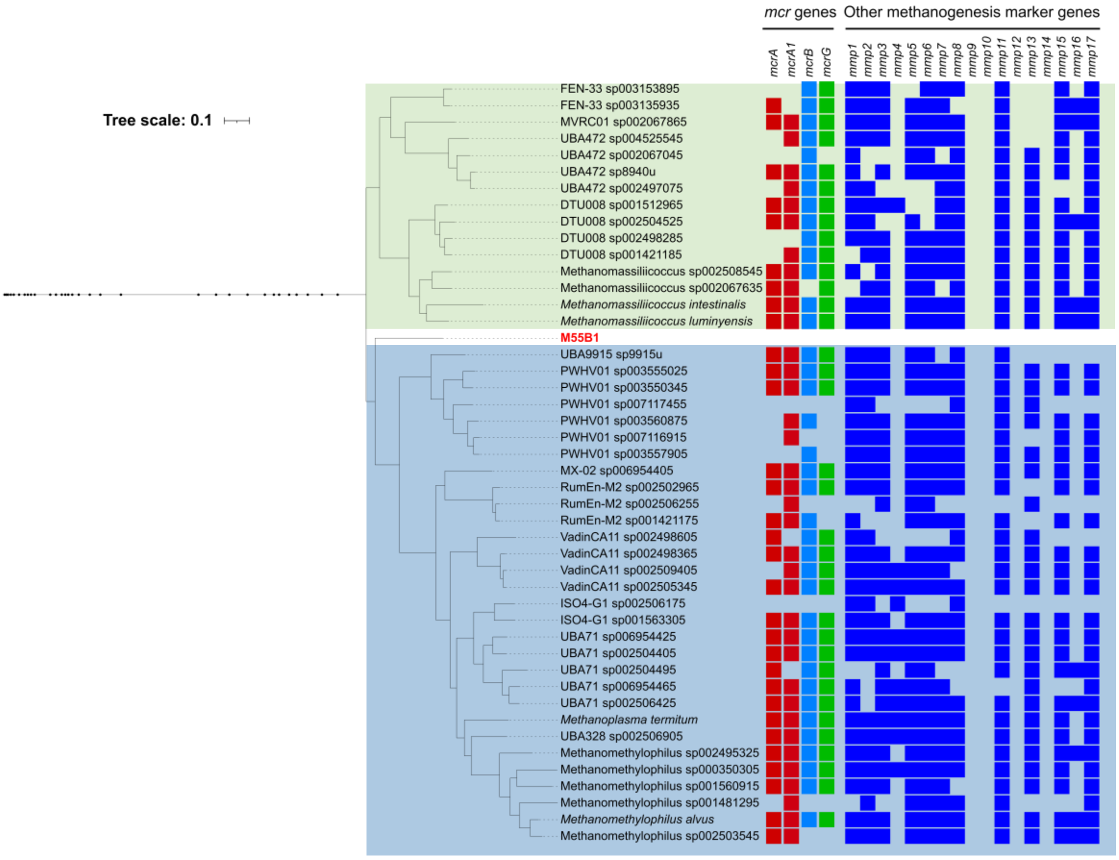

Supplementary Figure 2. Pruned phylogenomic tree of *Methanomassiliicoccales* and presence of methanogenesis marker genes in MAGs. The tree contains GTDB representative genomes of host-associated (highlighted in blue) and environmental clades (highlighted in green) in the *Methanomassiliicoccales* order. The genomes are annotated with Pfam and KEGG orthologous genes by EnrichM. The methanogenesis markers were selected according to Borrel *et al* (2014)[1,2]. KO numbers and Pfam IDs are listed in Table S2. The colored tiles indicate that the gene is present. Scale bar represents changes per amino acid site. Abbreviation: mmp, methanogenesis marker protein.

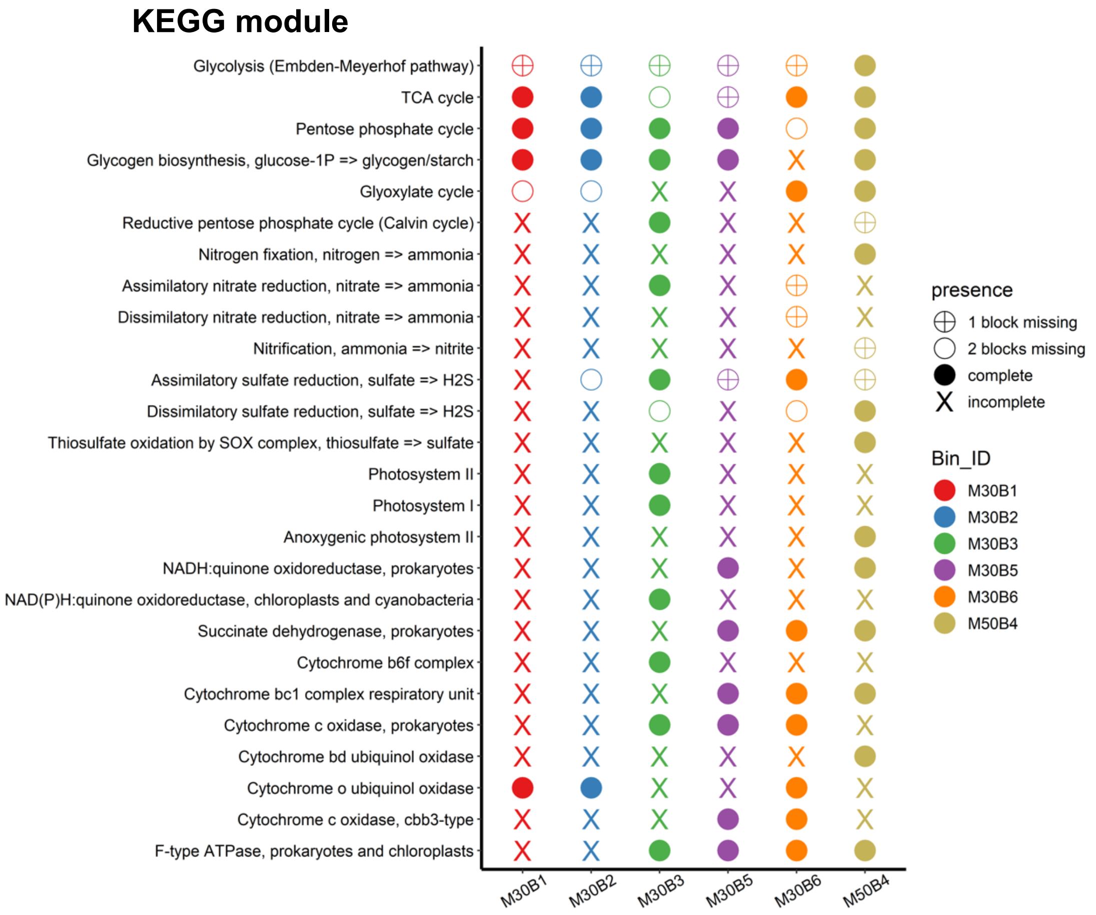

Supplementary Figure 3. Summary of metabolic features in the newly recovered complete MAGs.

The genomes are annotated with KEGG orthologues, and the completeness of the metabolic pathways is determined by EnrichM. The KEGG module numbers of each metabolic pathway are listed in Table S2. Each KEGG module is made of multiple functional units (blocks). Missing blocks represents missing gene(s) that form a functional unit. Incomplete modules indicate that more than two blocks are missing.

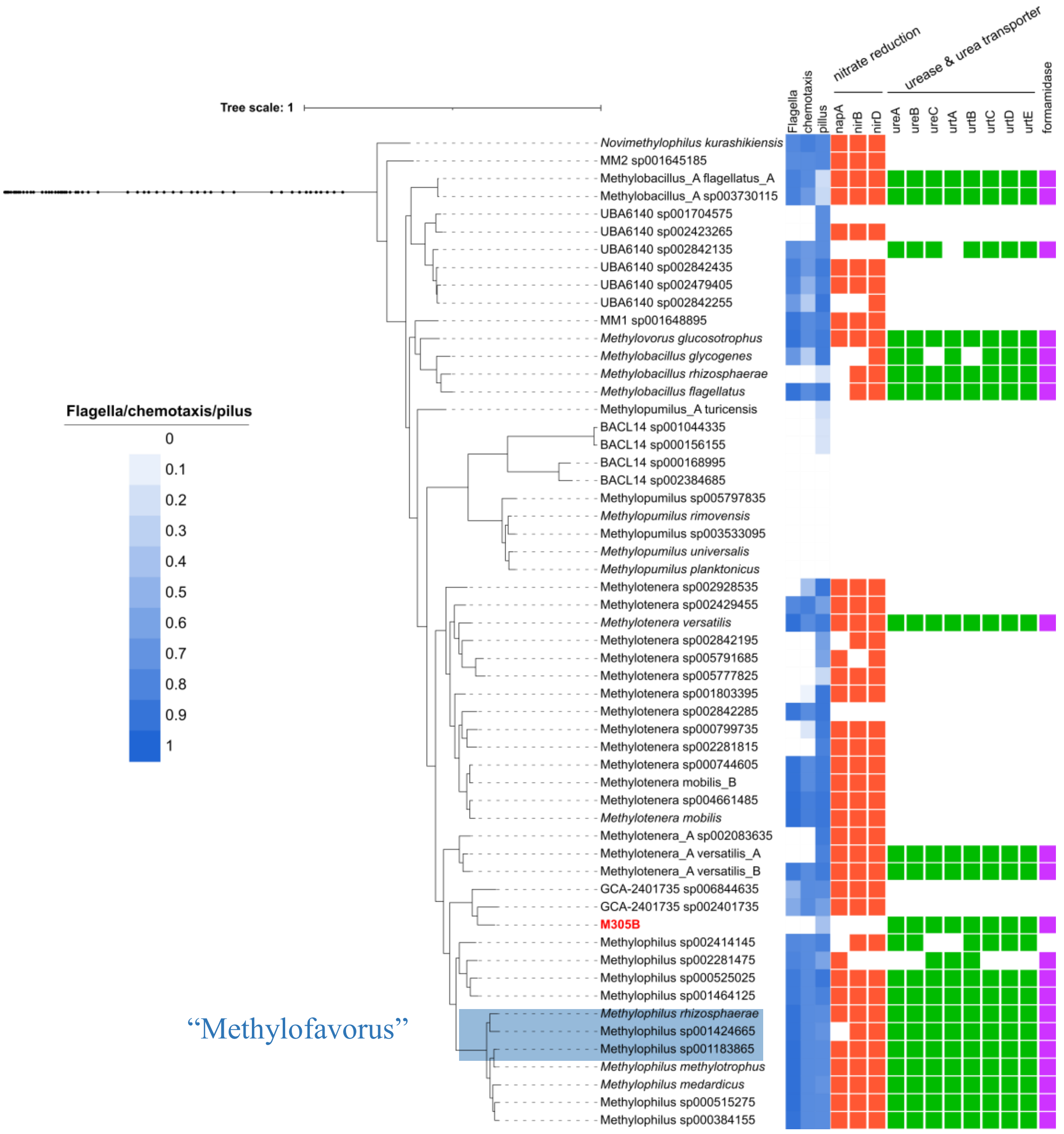

Supplementary Figure 4. Pruned phylogenomic tree of *Methylophilaceae*.

The tree contains GTDB representative genomes in the family *Methylophilaceae*. The genomes are annotated with Pfam and KEGG orthologous genes by EnrichM. The genes are selected according to Salcher *et al.*[3].KO numbers and Pfam IDs are listed in Table S3. The colored tiles indicate that the gene is present. The heatmap represents the proportions calculated by dividing the number of flagella, chemotaxis, and pilus-related genes found in the genome with related gene numbers in the KEGG database (list in Table S2). The scale bar represents changes per amino acid site.

Supplementary Table 1. Statistics on Nanopore and Illumina reads

	**3.0m**	**5.0m**	**5.5m**
	**Nanopore reads**		
Mean read length	2,063	1,000	7,006
Mean read quality	10.6	11.1	11.6
Median read length	761	472	3,669
Median read quality	11.1	11.3	11.9
Number of reads	6,705,136	12,569,375	683,928
Read length N50	5,578	1,775	13,545
Total bases	13,83 Gbp	12,57 Gbp	4,79 Gbp
Top 5 longest reads :			
**1.**	150,131	133,860	133,934
**2.**	132,395	130,047	120,861
**3.**	128,047	128,699	114,105
**4.**	121,232	123,796	114,062
**5.**	120,736	123,662	112,369
	**Illumina reads**		
Total reads (F+R)	486,655,848	579,511,508	509,594,810
Sequence length (bp)	45-100	45-100	45-100
Mean read length (F, R)	86, 91	91, 90	90, 93
Total bases	43.27 gbp	52.53 gbp	46.74 gbp
Q20 bases	42.12 gbp	52.53 gbp	45.82 gbp
Q30 bases	38.06 gbp	52.53 gbp	42.41 gbp

Supplementary Table 2. KEGG Orthology and Pfam families used in this study

Genes	Proteins/ functions	KEGG/TIGRFAMs #
**Methanogenesis**		
mcrA	Methyl-CoM reductase subunit A	K00399
mcrA2	Methyl-CoM reductase subunit A2	K00400
mcrB	Methyl-CoM reductase subunit beta	K00401
mcrG	Methyl-CoM reductase subunit G	K00402
mmp1	Methanogenesis marker protein 1	TIGR03266
mmp2	Methanogenesis marker protein 2	TIGR03267
mmp3	Methanogenesis marker protein 3	TIGR03268
mmp4	Methanogenesis marker protein 4	TIGR03270
mmp5	Methanogenesis marker protein 5	TIGR03271
mmp6	Methanogenesis marker protein 6	TIGR03272
mmp7	Methanogenesis marker protein 7	TIGR03274
mmp8	Methanogenesis marker protein 8	TIGR03275
mmp9	Methanogenesis marker protein 9	TIGR03277
mmp10	Methanogenesis marker protein 10 (putative methyl coenzyme M reductase-arginine methyltransferase)	TIGR03278
mmp11	Methanogenesis marker protein 11	TIGR03280
mmp12	Methanogenesis marker protein 12	TIGR03281
mmp13	Methanogenesis marker protein 13	TIGR03282
mmp14	Methanogenesis marker protein 14	TIGR03285
mmp15	Methanogenesis marker protein 15	TIGR03286
mmp16	Methanogenesis marker protein 16	TIGR03287
mmp17	Methanogenesis marker protein 17	TIGR03291
**Metabolic pathways**		
	Glycolysis (Embden-Meyerhof pathway)	M00001
	TCA cycle	M00009
	Pentose phosphate cycle	M00004
	Glycogen biosynthesis	M00854
	Glyoxylate cycle	M00012

	Reductive pentose phosphate cycle	M00165
	Nitrogen fixation	M00175
	Assimilatory nitrate reduction	M00531
	Dissimilatory nitrate reduction	M00530
	Nitrification, ammonia => nitrite	M00528
	Assimilatory sulfate reduction, sulfate => H2S	M00176
	Dissimilatory sulfate reduction, sulfate => H2S	M00596
	Thiosulfate oxidation by SOX complex	M00595
	Photosystem II	M00161
	Photosystem I	M00161
	Anoxygenic photosystem II	M00597
	NADH:quinone oxidoreductase, prokaryotes	M00144
	NAD(P)H:quinone oxidoreductase, chloroplasts and cyanobacteria	M00145
	Succinate dehydrogenase, prokaryotes	M00149
	Cytochrome b6f complex	M00162
	Cytochrome bc1 complex respiratory unit	M00151
	Cytochrome c oxidase, prokaryotes	M00155
	Cytochrome bd ubiquinol oxidase	M00153
	Cytochrome o ubiquinol oxidase	M00417
	Cytochrome c oxidase, cbb3-type	M00156
**Flagellum**		
flhG, fleN	flagellar biosynthesis protein FlhG	K04562
flhB2	flagellar biosynthesis protein	K04061
flgA	flagella basal body P-ring formation protein	K02386
flgB	flagellar basal-body rod protein FlgB	K02387
flgC	flagellar basal-body rod protein FlgC	K02388
flgD	flagellar basal-body rod modification protein	K02389
flgE	flagellar hook protein FlgE	K02390
flgF	flagellar basal-body rod protein FlgF	K02391
flgG	flagellar basal-body rod protein FlgG	K02392
flgH	flagellar L-ring protein precursor FlgH	K02393

flgI	flagellar P-ring protein precursor FlgI	K02394
flgJ	flagellar protein FlgJ	K02395
flgK	flagellar hook-associated protein 1 FlgK	K02396
flgL	flagellar hook-associated protein 3 FlgL	K02397
flgM	negative regulator of flagellin synthesis FlgM	K02398
flgN	flagella synthesis protein FlgN	K02399
flhA	flagellar biosynthesis protein FlhA	K02400
flhB	flagellar biosynthetic protein FlhB	K02401
flhC	flagellar transcriptional activator FlhC	K02402
flhD	flagellar transcriptional activator FlhD	K02403
flhF	flagellar biosynthesis protein FlhF	K02404
fliA	RNA polymerase sigma factor for flagellar operon	K02405
fliC	flagellin	K02406
fliD	flagellar hook-associated protein 2	K02407
fliE	flagellar hook-basal body complex protein FliE	K02408
fliF	flagellar M-ring protein FliF	K02409
fliG	flagellar motor switch protein FliG	K02410
fliH	flagellar assembly protein FliH	K02411
fliI	flagellum-specific ATP synthase [EC:3.6.3.14]	K02412
fliJ	flagellar FliJ protein	K02413
fliK	flagellar hook-length control protein FliK	K02414
fliL	flagellar FliL protein	K02415
fliM	flagellar motor switch protein FliM	K02416
fliNY, fliN	flagellar motor switch protein FliN/FliY	K02417
fliOZ, fliO	flagellar protein FliO/FliZ	K02418
fliP	flagellar biosynthetic protein FliP	K02419
fliQ	flagellar biosynthetic protein FliQ	K02420
fliR	flagellar biosynthetic protein FliR	K02421
fliS	flagellar protein FliS	K02422
fliT	flagellar protein FliT	K02423
flbT	flagellar protein FlbT	K06601
flaF	flagellar protein FlaF	K06602

flaG	flagellar protein FlaG	K06603
flaI	flagellar rod protein FlaI	K06604
**Chemotaxis**		
motA	chemotaxis protein MotA	K02556
motB	chemotaxis protein MotB	K02557
mcp	methyl-accepting chemotaxis protein	K03406
cheA	two-component system, chemotaxis family, CheA [EC:2.7.13.3]	K03407
cheW	purine-binding chemotaxis protein CheW	K03408
cheX	chemotaxis protein CheX	K03409
cheC	chemotaxis protein CheC	K03410
cheD	chemotaxis protein CheD [EC:3.5.1.44]	K03411
cheB	two-component system, chemotaxis family, response regulator CheB [EC:3.1.1.61]	K03412
cheY	two-component system, chemotaxis family, response regulator CheY	K03413
cheZ	chemotaxis protein CheZ	K03414
cheV	two-component system, chemotaxis family, response regulator CheV	K03415
**Pilus**		
pilA	type IV pilus assembly protein PilA	K02650
flp, pilA	pilus assembly protein Flp/PilA	K02651
pilB	type IV pilus assembly protein PilB	K02652
pilC	type IV pilus assembly protein PilC	K02653
pilD, pppA	leader peptidase (prepilin peptidase) / N-methyltransferase [EC:3.4.23.43 2.1.1.-]	K02654
pilE	type IV pilus assembly protein PilE	K02655
pilF	type IV pilus assembly protein PilF	K02656
pilG	twitching motility two-component system response regulator PilG	K02657
pilH	twitching motility two-component system response regulator PilH	K02658

pilI	twitching motility protein PilI	K02659
pilJ	twitching motility protein PilJ	K02660
pilK	type IV pilus assembly protein PilK	K02661
pilM	type IV pilus assembly protein PilM	K02662
pilN	type IV pilus assembly protein PilN	K02663
pilO	type IV pilus assembly protein PilO	K02664
pilP	type IV pilus assembly protein PilP	K02665
pilQ	type IV pilus assembly protein PilQ	K02666
pilR, pehR	two-component system, NtrC family, response regulator PilR	K02667
pilS, pehS	two-component system, NtrC family, sensor histidine kinase PilS [EC:2.7.13.3]	K02668
pilT	twitching motility protein PilT	K02669
pilU	twitching motility protein PilU	K02670
pilV	type IV pilus assembly protein PilV	K02671
pilW	type IV pilus assembly protein PilW	K02672
pilX	type IV pilus assembly protein PilX	K02673
pilY1	type IV pilus assembly protein PilY1	K02674
pilY2	type IV pilus assembly protein PilY2	K02675
pilZ	type IV pilus assembly protein PilZ	K02676
**nitrate reductase**		
napA	nitrate reductase	K02567
nirB	nitrite reductase	K00362
nirD	nitrite reductase (NADH) small subunit	K00363
**Urease and urea transporter**		
ureA	urease subunit gamma	K01430
ureB	urease subunit beta	K01429
ureC	urease subunit alpha	K01428
urtA	urea transport system substrate-binding protein	K11959
urtB	urea transport system permease protein	K11960
urtC	urea transport system permease protein	K11961
urtD	urea transport system ATP-binding protein	K11962

urtE	urea transport system ATP-binding protein	K11963
**Others**		
formamidase	formamidase	K01455

Supplementary References

1 Borrel, G. *et al.* Comparative genomics highlights the unique biology of Methanomassiliicoccales, a Thermoplasmatales-related seventh order of methanogenic archaea that encodes pyrrolysine. *Bmc Genomics* **15**, doi:Artn 679
10.1186/1471-2164-15-679 (2014).

2 Zinke, L. A. *et al.* Evidence for non-methanogenic metabolisms in globally distributed archaeal clades basal to the Methanomassiliicoccales. *Environ Microbiol* **23**, doi:10.1111/1462-2920.15316 (2021).

3 Salcher, M. M., Schaefle, D., Kaspar, M., Neuenschwander, S. M. & Ghai, R. Evolution in action: habitat transition from sediment to the pelagial leads to genome streamlining in Methylophilaceae. *Isme Journal* **13**, 2764-2777, doi:10.1038/s41396-019-0471-3 (2019).
