# Supplementary material for: Salvaging high-quality genomes of microbial species from a meromictic lake using a hybrid sequencing approach
Source: Commun Biol. 2021 Aug 23;4:996. doi: 10.1038/s42003-021-02510-6 (PMC8382752; doi:10.1038/s42003-021-02510-6)
Supplement: Supplementary file 3 — Description of Supplementary Files [file 42003_2021_2510_MOESM3_ESM.pdf]

**Description of Additional Supplementary Files**

**File name**: Supplementary Data 1
**Description:** Genome qualities and statistics evaluated by CheckM.

**File name**: Supplementary Data 2
**Description:** Taxonomy inference of bins by GTDB-Tk.
