# Supplementary material for: Salvaging high-quality genomes of microbial species from a meromictic lake using a hybrid sequencing approach
Source: Commun Biol. 2021 Aug 23;4:996. doi: 10.1038/s42003-021-02510-6 (PMC8382752; doi:10.1038/s42003-021-02510-6)
Supplement: Supplementary file 6 — Reporting Summary [file 42003_2021_2510_MOESM6_ESM.pdf]

nature research

Corresponding author(s): Sen-Lin Tang
Last updated by author(s): Jul 23, 2021

Reporting Summary

Nature Research wishes to improve the reproducibility of the work that we publish. This form provides structure for consistency and transparency in reporting. For further information on Nature Research policies, see our Editorial Policies and the Editorial Policy Checklist.

Statistics

For all statistical analyses, confirm that the following items are present in the figure legend, table legend, main text, or Methods section.

n/a	Confirmed	
☒	☐	The exact sample size (*n*) for each experimental group/condition, given as a discrete number and unit of measurement
☒	☐	A statement on whether measurements were taken from distinct samples or whether the same sample was measured repeatedly
☒	☐	The statistical test(s) used AND whether they are one- or two-sided<br>*Only common tests should be described solely by name; describe more complex techniques in the Methods section.*
☒	☐	A description of all covariates tested
☒	☐	A description of any assumptions or corrections, such as tests of normality and adjustment for multiple comparisons
☒	☐	A full description of the statistical parameters including central tendency (e.g. means) or other basic estimates (e.g. regression coefficient) AND variation (e.g. standard deviation) or associated estimates of uncertainty (e.g. confidence intervals)
☒	☐	For null hypothesis testing, the test statistic (e.g. *F*, *t*, *r*) with confidence intervals, effect sizes, degrees of freedom and *P* value noted<br>*Give P values as exact values whenever suitable.*
☒	☐	For Bayesian analysis, information on the choice of priors and Markov chain Monte Carlo settings
☒	☐	For hierarchical and complex designs, identification of the appropriate level for tests and full reporting of outcomes
☒	☐	Estimates of effect sizes (e.g. Cohen's *d*, Pearson's *r*), indicating how they were calculated

Our web collection on statistics for biologists contains articles on many of the points above.

Software and code

Policy information about availability of computer code

Data collection	NA
Data analysis	The workflow based on snakemake is available at https://github.com/SeanChenHCY/metaLAS

For manuscripts utilizing custom algorithms or software that are central to the research but not yet described in published literature, software must be made available to editors and reviewers. We strongly encourage code deposition in a community repository (e.g. GitHub). See the Nature Research guidelines for submitting code & software for further information.

Data

Policy information about availability of data

All manuscripts must include a data availability statement. This statement should provide the following information, where applicable:

- Accession codes, unique identifiers, or web links for publicly available datasets
- A list of figures that have associated raw data
- A description of any restrictions on data availability

All sequencing data and assembled genomes are available through National Center for Biotechnology Information (NCBI) repositories under BioProject ID: PRJNA721826. Sequence reads of metagenomes from samples at 3.0, 5.0, and 5.5 m deep can be found under SRA accession numbers SRR14300307, SRR14300308, SRR14300309, SRR14307495 , SRR14307795, and SRR14307796.

Field-specific reporting

Please select the one below that is the best fit for your research. If you are not sure, read the appropriate sections before making your selection.

☐ Life sciences ☐ Behavioural & social sciences ☒ Ecological, evolutionary & environmental sciences

For a reference copy of the document with all sections, see nature.com/documents/nr-reporting-summary-flat.pdf

Ecological, evolutionary & environmental sciences study design

All studies must disclose on these points even when the disclosure is negative.

Study description	Water samples at 3.0, 5.0, and 5.5 m deep were collected from Lake Shunet (54° 25'N, 90° 13'E) on July 21, 2010. Metagenomics study was performed with the samples/
Research sample	Salvaging complete and high-quality genomes of novel microbial species from a meromictic lake
Sampling strategy	NA
Data collection	DNA was sent to the NGS High Throughput Genomics Core at Biodiversity Research Center, Academia Sinica for Nanopore sequencing.
Timing and spatial scale	Water samples at 3.0, 5.0, and 5.5 m deep were collected from Lake Shunet (54° 25'N, 90° 13'E) on July 21, 2010.
Data exclusions	NA
Reproducibility	NA
Randomization	NA
Blinding	NA

Did the study involve field work? ☒ Yes ☐ No

Field work, collection and transport

Field conditions	The pH was 8.1, 7.6, and 6.7; water temperature was 15.5, 9.5, and 7.5°C; and salinity was 26, 40, 71g/L in the 3.0-, 5.0-, and 5.5-m samples, respectively.
Location	3.0, 5.0, and 5.5 m depth from Lake Shunet (54° 25'N, 90° 13'E)
Access & import/export	NA
Disturbance	NA

Reporting for specific materials, systems and methods

We require information from authors about some types of materials, experimental systems and methods used in many studies. Here, indicate whether each material, system or method listed is relevant to your study. If you are not sure if a list item applies to your research, read the appropriate section before selecting a response.

Materials & experimental systems

n/a	Involved in the study
☒	☐ Antibodies
☒	☐ Eukaryotic cell lines
☒	☐ Palaeontology and archaeology
☒	☐ Animals and other organisms
☒	☐ Human research participants
☒	☐ Clinical data
☒	☐ Dual use research of concern

Methods

n/a	Involved in the study
☒	☐ ChIP-seq
☒	☐ Flow cytometry
☒	☐ MRI-based neuroimaging
